# Controlled Heat Stress Promotes Myofibrillogenesis during Myogenesis

**DOI:** 10.1371/journal.pone.0166294

**Published:** 2016-11-08

**Authors:** Qiongyu Guo, Devin Miller, Hongying An, Howard Wang, Joseph Lopez, Denver Lough, Ling He, Anand Kumar

**Affiliations:** 1 Department of Plastic and Reconstructive Surgery, Johns Hopkins University School of Medicine, Baltimore, Maryland, United States of America; 2 Divisions of Metabolism and Endocrinology, Department of Pediatrics, Johns Hopkins University School of Medicine, Baltimore, Maryland, United States of America; University of Minnesota Medical Center, UNITED STATES

## Abstract

Hyperthermia therapy has recently emerged as a clinical modality used to finely tune heat stress inside the human body for various biomedical applications. Nevertheless, little is known regarding the optimal timing or temperature of heat stress that is needed to achieve favorable results following hyperthermia therapy for muscle regeneration purposes. The regeneration of skeletal muscle after injury is a highly complex and coordinated process that involves a multitude of cellular mechanisms. The main objective of this study was to characterize the effects of hyperthermal therapy on the overall behavior of myoblasts during myogenic differentiation. Various cellular processes, including myogenesis, myofibrillogenesis, hypertrophy/atrophy, and mitochondrial biogenesis, were studied using systematic cellular, morphological, and pathway-focused high-throughput gene expression profiling analyses. We found that C2C12 myoblasts exhibited distinctive time and temperature-dependence in biosynthesis and regulatory events during myogenic differentiation. Specifically, we for the first time observed that moderate hyperthermia at 39°C favored the growth of sarcomere in myofibrils at the late stage of myogenesis, showing universal up-regulation of characteristic myofibril proteins. Characteristic myofibrillogenesis genes, including heavy polypeptide 1 myosin, heavy polypeptide 2 myosin, alpha 1 actin, nebulin and titin, were all significantly upregulated (p<0.01) after C2C12 cells differentiated at 39°C over 5 days compared with the control cells cultured at 37°C. Furthermore, moderate hyperthermia enhanced myogenic differentiation, with nucleus densities per myotube showing 2.2-fold, 1.9-fold and 1.6-fold increases when C2C12 cells underwent myogenic differentiation at 39°C over 24 hours, 48 hours and 72 hours, respectively, as compared to the myotubes that were not exposed to heat stress. Yet, atrophy genes were sensitive even to moderate hyperthermia, indicating that strictly controlled heat stress is required to minimize the development of atrophy in myotubes. In addition, mitochondrial biogenesis was enhanced following thermal induction of myoblasts, suggesting a subsequent shift toward anabolic demand requirements for energy production. This study offers a new perspective to understand and utilize the time and temperature-sensitive effects of hyperthermal therapy on muscle regeneration.

## Introduction

Skeletal muscle accounts for 40% of total body mass and demonstrates an innate self-repair capability in response to minor tissue damage or injury [1, 2]. However, regenerating muscle tissues elements capable of spanning segmental muscle gaps or defects following severe injury remains a clinical challenge [3]. Recently, hyperthermal therapy has attracted increasing attention in the fields of tissue engineering and cancer chemo-therapeutics due to its potential to modify the extracellular microenvironment, and thus regulate localized tissue responses including immunological reaction, tissue perfusion, and tissue oxygenation [4, 5]. Although controlled thermal delivery of heat has shown some beneficial effects on myogenesis during skeletal muscle repair in both in vitro [6–8] and in vivo studies [9–11], the detailed and coordinated effects of thermal treatment on muscle regeneration remain under characterized, limiting the development of a tailored hyperthermia treatment protocol for muscle regeneration.

Skeletal muscle provides structural support and controls motor movements through highly organized long, tubular muscular cells or myofibers. Myofibers contain contractile fibril structures known as myofibrils that are composed of repeating units of sarcomeres. Sarcomeres primarily consist of thick filaments of myosin, thin filaments of actin, and elastic filaments of titin [12, 13]. Myofibrillogenesis, the development of the myofibril during myogenesis, plays a critical role in controlling the contractile strength of skeletal muscles [14, 15]. Recently, Yamaguchi et al. [6] and Oishi et al. [9] reported a fast-to-slow fiber-type shift in myotubes or myofibers during myogenesis in their in vitro and in vivo studies, respectively. Yet, their work solely focused on analyzing the expressions of myosin heavy chains. The effect of heat stress on myofibrillogenesis, including the expressions of various structural and regulatory proteins assembled in sarcomeres other than myosin such as actin, titin, and titin complexes, remains under characterized to date. Further investigation into thermal therapy applications on these fundamental functional proteins and resulting myogenic ultrastructure is of great importance to understanding temperature-induced alterations in muscle regeneration.

Myogenesis involves the orchestration of multiple biological processes including myofibrillogenesis, the hypertrophy/atrophy of cellular entities as well as mitochondrial biogenesis, all of which are critical to the development of proper muscular function. Myocytic hypertrophy is associated with a mass increase of myofibers through stimulating protein synthesis, whereas atrophy is related to protein breakdown through activating protein degradation pathways [16]. Mitochondrial biogenesis, while not only coupled with myogenesis through targeting key myogenic differentiation regulatory factors such as myogenin, may also be induced by environmental stimuli such as heat stress [17]. Current reports on the effects of controlled heat stress on hypertrophy/atrophy and mitochondrial biogenesis remain limited in that such methods have only utilized a single set temperature while practicing hyperthermal applications.

Understanding that the effect of controlled heat stress on skeletal muscle regeneration cannot be studied in isolation, the main objective of this study is to investigate the effects of controlled heat stress on overall biological behavior of myoblasts during myogenic differentiation, including myogenesis, myofibrillogenesis, hypertrophy/atrophy, and mitochondrial biogenesis. We hypothesize that investigating the time and temperature-dependences of these interrelated biological processes on heat treatment may provide valuable insight into the development of new applications for hyperthermal therapy in both muscle repair and regeneration efforts. In this study, a murine C2C12 myoblast cell line was used due to its morphological similarity to the primary myoblasts and its purity without contamination from other cell types [18]. We performed *in vitro* myogenic differentiation of the C2C12 cells at two elevated temperatures that can be reached in human physiological systems. These temperature values were defined as mild hyperthermia temperature at 39°C and severe hyperthermia temperature at 41°C. Here, we quantitatively examined and compared myotube morphology in terms of myotube width, myotube length, myotube density, nucleus density per myotube, and overall nuclei density. Additionally, we evaluated the effect of hyperthermia treatment on key gene expressions profiles that are associated with myogenesis, myofibrillogenesis, hypertrophy/atrophy, and mitochondrial biogenesis using a pathway-focused high-throughput gene array platform.

## Materials and Methods

### Myoblast culture and heat treatment

Murine C2C12 cells were purchased from ATCC (Manassas, VA). The cells were cultured on 6 well tissue culture plates, which was coated with 0.1% (w/v) type I bovine collagen solution (Life Technologies, Grand Island, NY) dissolved in 0.1 M acetic acid (Sigma, St. Louis, MO) over two hours at room temperature and rinsed with phosphate buffered saline three times before use. The cells were plated at a concentration of 2000 cells/cm^2^ and incubated at 37°C and 5% CO_2_ until reaching 80% confluence using skeletal muscle cell growth medium (Zenbio, Research Triangle Park, NC) containing Dulbecco's modified eagle medium (DMEM), fetal bovine serum, bovine serum albumin, fetuin, human epidermal growth factor, dexamethasone, human insulin, and penicillin. Myogenic differentiation of C2C12 myoblasts was then induced *in vitro* at three distinct ambient temperatures: 37°C, 39°C and 41°C using skeletal muscle cell differentiation medium (Zenbio, Research Triangle Park, NC) per the manufacturer’s instructions included within the kit.

### Immunofluorescence

Myotube formation and cellular morphologies were examined following staining for myosin heavy chain at various myogenic differentiation time points of 0 h, 24 h, 48 h, 72 h and 5 d. Muscle cell cultures were fixed with 4% paraformaldehyde (Affymetrix Life Sciences) followed by permeabilization with 0.2% Triton X-100 (Sigma) in phosphate buffered saline. Blocking was performed using 1% bovine serum albumin (Sigma). Cultures were then incubated with mouse anti-myosin heavy chain antibody conjugated with Alexa Fluor 488 (5 ug/ml, eBioscience, San Diego, CA) for 1 hour at room temperature and counterstained with Hoechst 33342 nucleic acid stain (Invitrogen) at 1:1000. Image acquisition was performed with an inverted fluorescence microscope (Olympus MP1000, Center Valley, PA).

### Quantification of cellular morphology

Myotube morphologies, including myotube width, myotube length, myotube density, nucleus density per myotube, and total nucleus density, were measured using ImageJ software (NIH, Bethesda, MD). The cells of each condition were cultured in three separate wells. Microscopic images of three fields in each well were randomly captured for morphology analyses. Myotube width and length of each condition were determined from five longest myosin heavy chain-positive myotubes. Nucleus density per myotube was also obtained from the five longest myotubes. Myotube and total nucleus densities and their standard deviations of each condition were calculated from the microscopic images of three separate wells.

### Gene expression analysis

Total RNA was isolated from cultured cells using TRIzol Plus RNA purification kit (Life Technologies) and reverse transcribed into cDNA using RT^2^ first strand kit (Qiagen, Valencia, CA). cDNA was tested using a RT^2^ profiler PCR array kit (mouse skeletal muscle: myogenesis and myopathy, Qiagen). Real-time PCR reactions were performed using a CFX Connect Real-Time System (Bio-Rad, Hercules, CA). The PCR primers are listed in **S1 Table** (UniGene database, www.ncbi.nlm.nih.gov/unigene). Data were analyzed using a RT^2^ profiler PCR array software (Qiagen) and reference genes were automatically selected from the full plate using the software. Gene expression of all conditions was tested in duplicate. The relative gene expression was determined using a 2^-ΔΔC(T)^ method.

### Western blot analysis

Western blot analysis was carried out as described earlier [19]. Briefly, cultured cells were harvested in cell lysis buffer (Cell Signaling Technology) and denatured in SDS loading buffer. Protein (15 μg) of each sample was loaded for electrophoresis in 3–8% SDS PAGE and Ac-Tris gel (Life Technology). After wet transfer and blocking, immunoblots were incubated with primary antibodies at recommended dilution ratios at 4°C overnight. Primary antibodies include Myh1 (1:2500, clone NOQ7.5.4D, anti-slow skeletal myosin heavy chain antibody, Abcam), Myh2 (1:500, clone MY32, anti-fast skeletal myosin heavy chain antibody, Abcam), and GAPDH (1:5000, clone 6C5, Abcam). The next day the blots were incubated with second antibody (Santa Cruz) 1:5000 at room temperature and finally detected with ECL reagent (GE Healthcare).

### Statistics

Data were represented as averages ± standard deviations, unless otherwise specified. Two-tailed unpaired *t*-tests were performed to determine significance using Graphpad Prism (Graphpad Software Version 6, Graphpad Software, Inc., La Jolla, CA). *p* value of <0.05 was considered statistically significant.

## Results

### Early moderate hyperthermia improves myotube formation and morphology

**Fig 1:**
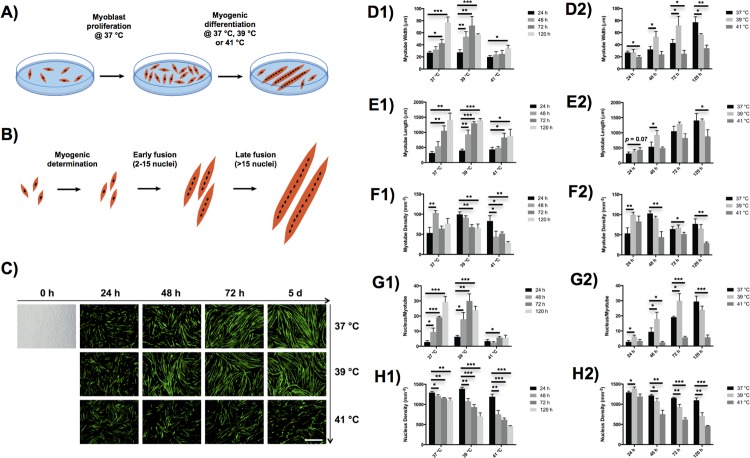
Effects of hyperthermia treatment on myotube morphology during myogenic differentiation of C2C12 myoblasts. Schematics of (A) hyperthermia performed on myogenic differentiation after myoblast proliferation, and (B) myoblasts undergoing three steps, i.e. myogenic determination, early fusion, and late fusion, during myogenic differentiation. (C) Characteristic microscopy images of myoblasts in bright field (0 h) and myosin heavy chain-positive myotubes (green). The myotube images were taken at four different time periods, i.e. 24 h, 48 h, 72 h, and 120 h, and three different temperatures, i.e. 37°C, 39°C, and 41°C. Scale: 500 μm. (D-H) Myotube morphologies, including myotube width (D), myotube length (E), myotube density (F), nucleus density per myotube (G), and overall nucleus density (H), were quantitatively analyzed. The error bars in (D-H) represent the standard deviation of the mean. Myotubes differentiated over 24 h were tested as a control in (D1, E1, F1, G1 and H1), and the myotubes cultured at 37°C were used as a control in (D2, E2, F2, G2 and H2). All statistical differences against the controls are indicated. *p < 0.05, **p < 0.01, ***p < 0.001.

Time and temperature-dependence of controlled heat stress on myotube morphology during myogenesis were systematically investigated *in vitro*. As depicted in Fig 1A, C2C12 myoblasts were cultured in proliferative media until reaching 80% confluence, and then myogenically induced at three distinct temperatures, including human physiological temperature at 37°C and two elevated temperatures at 39°C and 41°C, for up to 5 d. Myotubes undergoing early fusion stage are defined as those containing less than or equal to 15 nuclei per myotube, and the myotubes in late fusion stage are defined as those containing more than 15 nuclei per myotube (Fig 1B) [20, 21]. Typical morphologies of myotubes differentiated at different temperatures for various time points are shown in Fig 1C.

The early and late myotube formation processes were quantitatively examined, including myotube width, myotube length, myotube density, nucleus density per myotube, and overall nuclei density (Fig 1D–1H). Specifically, as compared to the control cells cultured at 37°C, the width of the myotubes incubated at 39°C first significantly increased after differentiated from 48 h to 72 h, but then remarkably decreased after 5 d of incubation (Fig 1D). In contrast, the myotubes cultured at 41°C significantly decreased in width compared with myotubes that were not heat-stressed. The length of myotubes exhibited different temperature dependence over the differentiation process (Fig 1E). Similar to the control culture at 37°C, myotube length continuously increased with differentiation time at both elevated temperatures. The length of myotubes increased with temperature at the very early time point of 24 h, and then showed a different trend at later time points with the cells cultured at 39°C forming the longest myotubes. Similarly, the density of myotubes differentiated at 41°C was slightly higher than that at 37°C after 24 h of differentiation, but became lower than that at 37°C at later time points (Fig 1F).

An analysis of nucleus density per myotube clearly demonstrated that myoblasts cultured at 39°C entered the late myotube formation process earlier than the control cells cultured at 37°C, whereas the myoblasts exposed to heat stress at 41°C failed to enter the late myotube formation process even after differentiated over 5 d (Fig 1G). The nucleus densities per myotube showed 2.2-fold, 1.9-fold, and 1.6-fold increases when C2C12 cells underwent myogenic differentiation at 39°C over 24 h, 48 h and 72 h, respectively, as compared to the controlled cells differentiated at 37°C. Nevertheless, the long period of mild heat stimulation at 39°C over 5 d did not further increase nucleus density per myotube compared with shorter time period of heat stress treatment. Lastly, the overall density of nuclei of both myotubes and myoblasts gradually decreased with time at all three temperatures, but dropped down more quickly at elevated temperatures (Fig 1H). Therefore, a moderately elevated temperature of 39°C, but not as high as 41°C, promoted myogenesis during the early myotube formation process until 3 d in terms of myotube width and nucleus density per myotube. At very early time points, myoblasts treated at a temperature of 41°C still exhibited some beneficial effects on promoting myogenesis in terms of myotube length and density.

### Early hyperthermia mediates myogenic determination but only moderate hyperthermia promotes long-term myogenic differentiation

**Fig 2:**
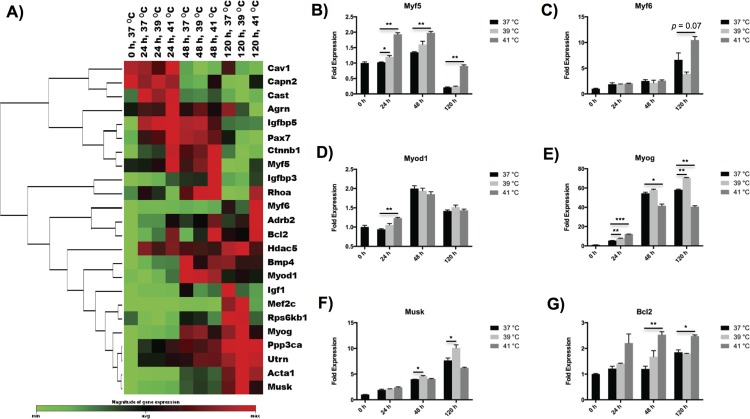
Effects of hyperthermia treatment on myogenesis of C2C12 myoblasts. (A) Clustergram of myogenesis genes showing time- and temperature-dependences on hyperthermia treatment during myogenic differentiation. (B-G) Characteristic myogenesis genes represented in bar figures. The error bars in (B-G) represent the standard deviation of the mean, and the myotubes cultured at 37°C were used as a control. All statistical differences against the control are indicated. *p < 0.05, **p < 0.01, ***p < 0.001.

To further examine the influence of heat stimulation on myogenesis, we analyzed gene expression levels of myotubes using a pathway-focused RT-PCR profiler array (Fig 2). Myogenic regulatory factors (MRFs), including Myf5, Myf6, Myod1 and Myog, are important myogenesis genes and play a critical role in regulating the differentiation of myoblasts. Among them, Myf5, Myf6 and Myod1 are myogenic determination factors, and Myog is a myogenic differentiation factor [22]. We found that the expression of Myf5 was highest at 41°C throughout the differentiation process, whereas Myf6 showed the highest response at 41°C only after the cells had differentiated for 5 d (Fig 2B and 2C). Myod1 was most highly expressed after 24 h of differentiation at 41°C, but did not show any significant differences at later time points (Fig 2D). Similarly, the gene expression level of Myog only transiently up-regulated upon exposure to heat stimulation at 41°C after 24 h of differentiation, but remarkably decreased at later time points (Fig 2E). Instead, the highest response of Myog was observed at 39°C at the latter phase of myogenesis (48 h to 5 d).

In addition, myotubes also exhibited distinct behaviors in other myogenesis-associated genes upon exposure to different levels of heat stress. We observed the peak mRNA of Mef2c, the myogenic transcription factor directing terminal differentiation of myoblasts into myotubes [23], after mild heat treatment at 39°C over 5 d. Similarly, the gene Musk, a muscle-specific receptor tyrosine kinase important for neuromuscular junction [24], showed the highest expression following heat exposure at 39°C over 48 h up to 5 d (Fig 2F). In contrast, severe hyperthermia at 41°C exhibited more complex effect on myogenesis genes. Gene expression of Igfbp3, an insulin-like growth factor binding protein that supports myogenic differentiation [25], was only boosted upon severe heat stimulation at 41°C over the whole process of myogenic differentiation compared with the control cells cultured at 37°C. The gene Ctnnb1, a β-catenin supporting skeletal muscle regeneration [26], exhibited the highest expression at 41°C after 24 h to 48 h of differentiation, but showed the lowest expression at 41°C after differentiated over 5 d. The gene Pax7 that is restricted to undifferentiated cells only demonstrated the highest expression at 41°C at the very early time point of 24 h [22]. Interestingly, the gene Rhoa, which is rapidly and transiently increased during myogenic determination, but quickly decreased during myogenic fusion [27], showed significantly higher expression at 41°C than the other two temperatures after 5 d of differentiation, indicating that myogenic fusion was inhibited at the late stage of myogenic differentiation under severe hyperthermia condition. Finally, the gene Bcl2, an important gene regulating cellular apoptosis [28], showed increased expression with incubation temperatures over the myogenic differentiation process (Fig 2G), which is consistent with our finding in the overall nucleus density (Fig 1H). Some important myogenesis genes, which are also associated with muscle myofibrillogenesis or hypertrophy/atrophy such as Acta1 and Capn2, were found to be affected by hyperthermia conditions and will be discussed in the following sections.

### Moderate hyperthermia enhances myofibrillogenesis during myogenic differentiation

**Fig 3:**
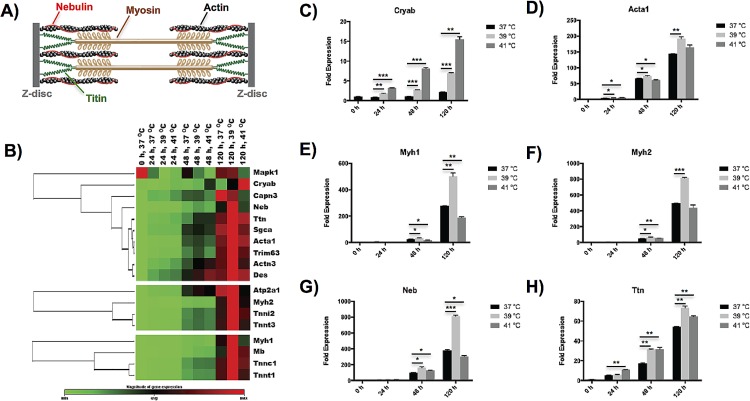
Effects of hyperthermia treatment on myofibrillogenesis of C2C12 myoblasts during myogenic differentiation. (A) Schematic of a sarcomere segment in myofibril containing various important proteins, including actin, myosin, nebulin and titin. (B) Clustergram of myofibrillogenesis genes, including genes associated with titin complex (top), fast-twitch fibers (middle), and slow-twitch fibers (bottom), showing time- and temperature-dependences on hyperthermia treatment during myogenic differentiation. (C-H) Characteristic myofibrillogenesis genes represented in bar figures. The error bars in (C-H) represent the standard deviation of the mean, and the myotubes cultured at 37°C were used as a control. All statistical differences against the control are indicated. *p < 0.05, **p < 0.01, ***p < 0.001.

Skeletal muscle contractility genes of differentiated myoblasts were analyzed in details by examining the expression of myofibrillogenesis genes, which include genes associated with titin complex, fast-twitch fibers, and slow-twitch fibers. As shown in Fig 3B, we for the first time observed that moderate hyperthermia at 39°C favored the growth of sarcomere in myofibrils at the late stage of myogenesis, showing universal up-regulation of characteristic myofibril genes. Characteristic myofibrillogenesis genes, including alpha 1 actin (Acta1) (Fig 3D), heavy polypeptide 1 myosin (Myh1) (Fig 3E), heavy polypeptide 2 myosin (Myh2) (Fig 3F), nebulin (Neb) (Fig 3G) and titin (Ttn) (Fig 3H), were all significantly upregulated (p < 0.01) after C2C12 cells differentiated at 39°C over 5 d as compared to the control cells cultured at 37°C. Moreover, the genes associated with fast-twitch fibers and slow-twitch fibers were all up-regulated at 39°C as compared with the other two temperatures after differentiated for 5 d [29] (Fig 3B). Note that only three titin-associated protein genes, i.e. Capn3, Cryab and Mapk1, showed different behaviors upon heat stimulation because of following reasons. The gene Capn3, whose overexpression has been found to negatively affect myogenic differentiation [30, 31], showed the highest expression at 37°C after 5 d of differentiation. Cryab, an important gene of small heat-shock-protein sensitive to heat stress [32], exhibited an incremented expression with temperature during myogenic differentiation (Fig 3C). The gene Mapk1, a positive regulator of myofibrillogenesis [33, 34], showed the highest expression at 37°C at the early stage of myogenic differentiation, and demonstrated an expression at 39°C comparable to that at 37°C after differentiated for 5 d.

The protein content of key myofibrillogenesis genes was validated in western blot analysis of C2C12 cells exposed to heat stress (S1 Fig). We found that the cells that underwent myogenic differentiation over 5 days demonstrated up-regulated Myh1 (clone NOQ7.5.4D, slow-twitch fiber) and Myh2 (clone MY32, fast-twitch fiber) upon mild heat treatment at 39°C compared with the cells either cultured at 37°C without heat stress or at 41°C with severe heat treatment. In addition, both Myh1 and Myh2 were undetectable in the control cells that were not differentiated (37°C, 0 h). These findings are consistent with our observations of the mRNA expression tested by RT-PCR (Fig 3).

### Hyperthermia performs a complex effect on hypertrophy/atrophy

**Fig 4:**
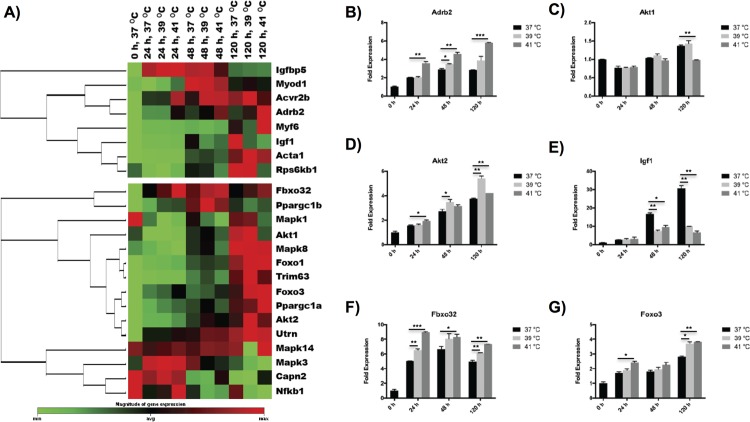
Effects of hyperthermia treatment on hypertrophy/atrophy of C2C12 myoblasts during myogenic differentiation. (A) Clustergram of hypertrophy (top) and atrophy (bottom) genes showing time- and temperature-dependences on hyperthermia treatment during myogenic differentiation. (B-G) Characteristic hypertrophy/atrophy genes represented in bar figures. The error bars in (B-G) represent the standard deviation of the mean, and the myotubes cultured at 37°C were used as a control. All statistical differences against the control are indicated. *p < 0.05, **p < 0.01, ***p < 0.001.

The heat stress treatment showed a comprehensive effect on hypertrophy/atrophy genes (Fig 4). For hypertrophy genes, we found that controlled heat stress mediated four important genes, i.e. Adrb2, Acta1, Akt1, Akt2, but suppressed the expression of the Igf1 gene. The adrenergic receptor, Adrb2, which supports hypertrophy and has anti-atrophy effects [16], demonstrated an increased expression with temperature after differentiated over 5 d (Fig 4B). In contrast, Acta1, a gene which mediates hypertrophy, exhibited the highest expression at 39°C during the whole differentiation process (Figs 3C and 4A). In addition, Akt1 and Akt2, key signaling proteins in the cellular pathways that mediate hypertrophy [35], were up-regulated in the highest level at 39°C at the late stage of differentiation (5 d) (Fig 4C and 4D). In contrast, Igf1, an important gene that can induce hypertrophy [16], was down-regulated at both 39°C and 41°C during myogenesis after 24 h of differentiation (Fig 4E). Furthermore, four important genes involved in muscle atrophy, i.e. Capn2, Fbxo32, Foxo1 and Foxo3, were up-regulated with heat stress treatment. Specifically, Capn2, which is involved in the initial degradation of myofibrillar proteins [31], was especially up-regulated at 41°C after 48 h of differentiation. Fbox32, which is normally expressed during muscle atrophy [36], showed an increased expression with temperature during the whole differentiation process (Fig 4F). Foxo1 and Foxo3, two members of the forkhead family of transcriptional activators that mediates muscle atrophy [37], were up-regulated at both 39°C and 41°C after 5 d of differentiation (Fig 4G).

### Hyperthermia induces mitochondrial biogenesis across all temperatures

**Fig 5:**
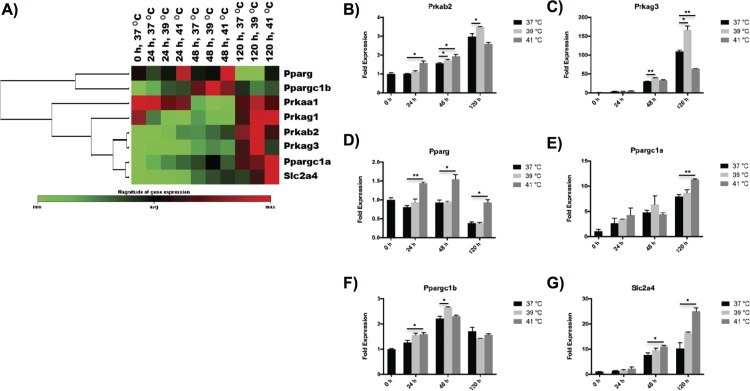
Effects of hyperthermia treatment on mitochondrial biogenesis of C2C12 myoblasts during myogenic differentiation. (A) Clustergram of mitochondrial biogenesis genes showing time- and temperature-dependences on hyperthermia treatment during myogenic differentiation. (B-G) Characteristic mitochondrial biogenesis genes represented in bar figures. The error bars in (B-G) represent the standard deviation of the mean, and the myotubes cultured at 37°C were used as a control. All statistical differences against the control are indicated. *p < 0.05, **p < 0.01, ***p < 0.001.

Mitochondrial biogenesis of differentiated myoblasts was also affected by hyperthermal treatment at different stages of myogenesis (Fig 5). Here, we examined four AMP-activated protein kinase (AMPK) genes (i.e. Prkaa1, Prkab2, Prkag1 and Prkag3), three peroxisome proliferator activated receptors (i.e. Pparg, Ppargc1a and Ppargc1b), and one glucose transporter (i.e. Slc2a4). Two AMPK genes, i.e. Prkab2 and Prkag3, demonstrated the highest expression at 39°C after 5 d of differentiation, while the other two AMPK genes did not show significant response on hyperthermia conditions (Fig 5B and 5C). The peroxisome proliferator activated receptor, Ppargc1b, expressed in the highest level at 39°C but at an earlier time point of 48 h (Fig 5F). Differently, the other three genes, Pparg, Ppargc1a and Slc2a4, exhibited the highest expression at 41°C after differentiated for 5 d (Fig 5D, 5E and 5G).

## Discussion

Translational hyperthermal techniques have recently been developed to deliver focal heat stress within selected tissue bodies for various clinical applications [38]. Skeletal muscle, the intrinsic heat-producing tissue of the human body, has demonstrated beneficial responses to controlled heat stress during times of regeneration. The repair of skeletal muscles involves various cellular responses that are highly synchronized and carefully regulated. However, a more comprehensive study of the influence of heat stress on muscle repair and regeneration, especially for the effect on the expression of various critical proteins other than heat shock proteins, is lacking [39, 40]. In this study, we quantitatively examined the comprehensive effects of controlled heat stress on the myogenic differentiation of C2C12 myoblasts in various regulatory events, including myogenesis, myofibrillogenesis, muscle hypertrophy/atrophy, and metabolism.

The temperature values targeted during hyperthermal therapy strongly influences the myogenic differentiation process of myoblasts. In this study, we observed a time and temperature-dependent hyperthermic effect on cellular fusion and subsequent myogenic differentiation of murine C2C12 cells. Although numerous studies reported beneficial effects of controlled heat stress on myogenic differentiation process of myoblasts [6, 8], we for the first time observed distinct impacts of severe heat treatment on myogenic determination factors compared with myogenic differentiation factors. Severe hyperthermia treatment at 41°C promoted myogenic determination factors (i.e. Myf5, Myf6 and Myod1) up to 5 d, it was only able to support the differentiation of myoblasts characterized by Myog at the very early time point of 24 h (Fig 2). Such results can be explained by the cytotoxicity associated with long-term severe hyperthermia treatment. In contrast, mild hyperthermia treatment at 39°C enhanced myogenic differentiation even at the late stage of differentiation. This is consistent with the change in apoptosis and heat shock gene expression that was observed (Figs 2G and 3C). Compared to the control culture at 37°C, both apoptosis and heat shock genes were strongly expressed at severe hyperthermia temperature of 41°C, whereas their expressions only slightly increased with moderate heat stress at 39°C. These gene expression results are consistent with what we observed in the cellular morphology study. The myoblasts treated at mild hyperthermia temperature promoted the myotube fusion speed, but those at severe hyperthermia temperature failed to enter the late myotube fusion process throughout the differentiation process (Fig 1). Therefore, heat treatment promotes the myogenic determination of myoblasts at the early stage of differentiation, while moderate heat stress is required to enhance the myogenic differentiation at the later stage of differentiation.

Gene expression of myofibrillogenesis, the generation of myofibrils that produce contractility in skeletal muscle, was for the first time found to be universally up-regulated by moderate hyperthermia. We analyzed three groups of genes associated with fast-twitch fibers, slow-twitch fibers and titin complex (Fig 3). Most genes initially showed low expression following heat treatment up to 48 h, but remarkably expressed at the late stage of myogenesis (5 d). Compared to the control temperature of 37°C and severe hyperthermia temperature of 41°C, moderate hyperthermia at 39°C exhibited the highest expression of almost all of the important protein components found in sarcomere, including myosin, actin, titin and nebulin (Fig 3A) [13]. Severe hyperthermia failed to promote myofibrillogenesis possibly due to cytotoxicity with overexpression of apoptosis and heat shock genes.

The genes associated with hypertrophy/atrophy demonstrated a mixed dependence on the differentiation temperature of myoblasts. Hyperthermia conditions promoted both hypertrophy and atrophy genes, indicating that heat stress mediates both protein synthesis and breakdown inside myotubes through different pathways. The competing effect of hypertrophy/atrophy genes may explain what we observed in the time- and temperature-dependences of myotube width during myogenic differentiation. Compared to the control temperature, moderate hyperthermia only significantly increased the myotube width up to 3 d, but quickly showed a decreased myotube width after differentiated over 5 d.

Gene expression of mitochondrial biogenesis was up-regulated by hyperthermia treatment, but showed different temperature-dependence on different pathways. Two genes associated with AMPK (Prkab2 and Prkag3), the key energy sensor in mitochondrial biogenesis [7], were expressed in the highest level under moderate hyperthermia at 39°C at the late stage of myogenesis (5 d, Fig 5B and 5C). In contrast, Ppargc1a, the master regulator of mitochondrial biogenesis [37], exhibited higher expression under severe hyperthermia at 41°C than the other two conditions (Fig 5E). Compared to Ppargc1a, Ppargc1b, another peroxisome proliferator receptor that controls mitochondrial capacity in an independent manner [7], demonstrated the highest expression under moderate hyperthermia at an earlier stage of myogenesis (48 h) (Fig 5F). On the other hand, the gene Slc2a4, an important glucose transporter regulated by Ppargc1a [41], exhibited similar behavior as Ppargc1a (Fig 5G). Consequently, the mitochondrial biogenesis, which provided energy production for myogenesis, was boosted by hyperthermia treatment, which is consistent with previous report published by Liu et al. [7]

In summary, during myogenic differentiation, myoblasts exhibited distinctive time- and temperature-dependences in different biosynthesis and regulatory events, including myogenesis, myofibrillogenesis, hypertrophy/atrophy, and mitochondrial biogenesis. During myogenesis, early hyperthermia (≤48 h) promoted myogenic determination of myoblasts, but only moderate hyperthermia enhanced myogenic differentiation of the cells. The myotubes under severe hyperthermia never entered the late stage of myogenic fusion process due to cytotoxicity resulted from overexpression of apoptosis and heat shock genes. Moderate hyperthermia also favored the growth of sarcomere in myofibril at the late stage of myogenesis, showing universal up-regulation of the characteristic genes of myofibril proteins. Yet, elevated temperature environment did not only promote protein synthesis inside myotubes, but also mediated protein breakdown during myogenesis. Therefore, hyperthermia treatment demonstrated a comprehensive effect on hypertrophy/atrophy genes and showed a favorable result in myotube size only at the early stage of myogenesis. Therefore, atrophy genes were sensitive even to moderate hyperthermia. Strictly controlled heat stress would be required to minimize the development of atrophy in myotubes, which may be achieved through hyperthermia treatment under lower temperature and shorter time periods in an intermittent manner. On the other hand, hyperthermia increased metabolic activities and mitochondrial biogenesis, producing more energy for the various regulatory events throughout myogenic differentiation.

## Conclusions

During skeletal muscle regeneration, myogenesis is accompanied with numerous regulatory and biosynthesis events, including myofibrillogenesis, hypertrophy/atrophy, and mitochondrial biogenesis, which strongly affect the final function of regenerated muscle. This study has demonstrated comprehensive effect of hyperthermia treatment on the myogenic differentiation of myoblasts through systematically analyzing characteristic gene expression associated with the various biosynthesis and regulatory events as well as cellular morphologies. Gene expression of myofibrillogenesis, the generation of myofibrils that produce contractility in skeletal muscle, was for the first time found to be universally up-regulated by moderate hyperthermia at 39°C. Furthermore, early application of hyperthermia up to 41°C promoted myogenic determination of myoblasts, but only moderate hyperthermia at 39°C enhanced myogenic differentiation. Yet, hyperthermia environment demonstrated a comprehensive effect on hypertrophy/atrophy genes and showed a significant increase in myotube dimension only at the early stage of myogenesis. This study provides novel insight to the impact of hyperthermal therapy on regenerative forms of muscle healing. Physical exercises, which are known to be able to raise muscle temperature up to 41°C [42], may be readily applied in a controlled manner to stimulate muscle repair processes.

## Supporting Information

S1 Fig Western blot analysis of myofibrillogenesis proteins in C2C12 cells exposed to heat stress. The cells underwent myogenic differentiation over 5 days and showed that the expression of Myh1 (clone NOQ7.5.4D, slow-twitch fiber) and Myh2 (clone MY32, fast-twitch fiber) were both up-regulated upon mild heat treatment at 39°C compared with the cells either cultured at 37°C without heat stress or at 41°C with severe heat treatment. As expected, both Myh1 and Myh2 were undetectable in the control cells that were not differentiated (37°C, 0 h).
(TIFF)

S1 Table RT-PCR primers analyzed in this study. (XLSX)
