# Supplementary figures and images for: Controlled Heat Stress Promotes Myofibrillogenesis during Myogenesis
Source: PLoS One. 2016 Nov 8;11(11):e0166294. doi: 10.1371/journal.pone.0166294 (PMC5100975; doi:10.1371/journal.pone.0166294)

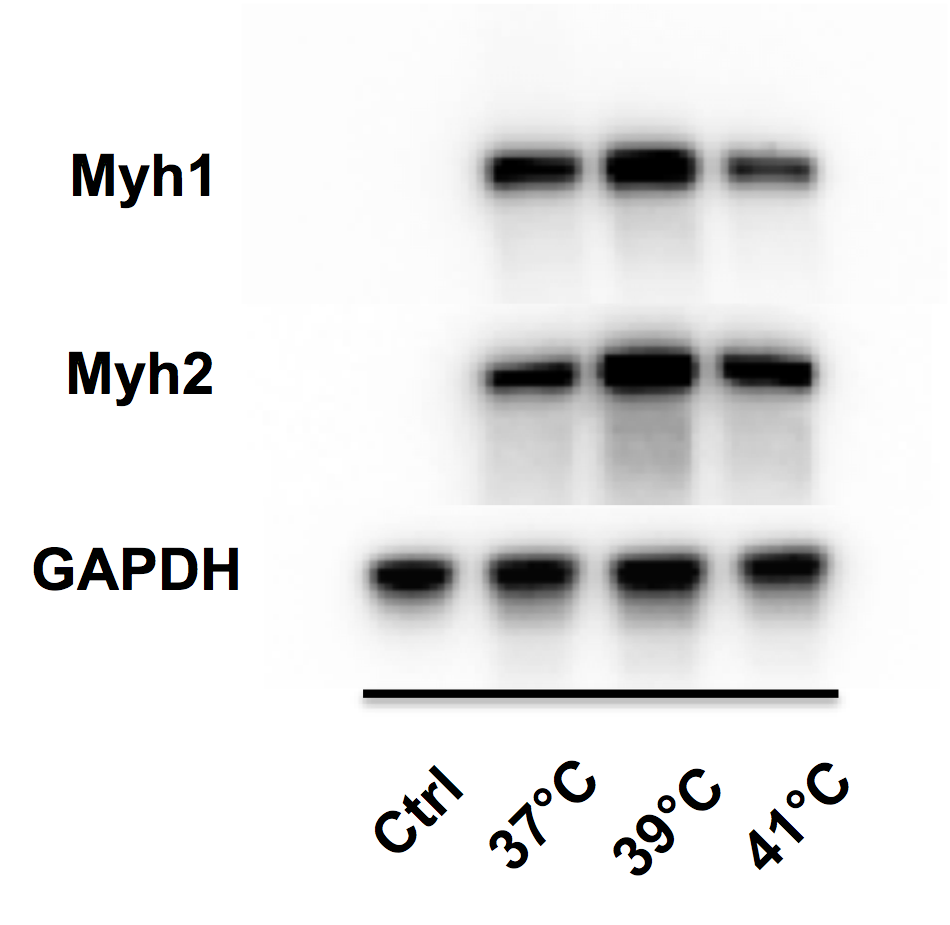

Supplement: S1 Fig — The cells underwent myogenic differentiation over 5 days and showed that the expression of Myh1 (clone NOQ7.5.4D, slow-twitch fiber) and Myh2 (clone MY32, fast-twitch fiber) were both up-regulated upon mild heat treatment at 39°C compared with the cells either cultured at 37°C without heat stress or at 41°C with severe heat treatment. As expected, both Myh1 and Myh2 were undetectable in the control cells that were not differentiated (37°C, 0 h). (TIFF) [file pone.0166294.s001.tiff]
